# Mediating Effect of Motor Competence on the Relationship between Physical Activity and Quality of Life in Children with Attention Deficit Hyperactivity Disorder

**DOI:** 10.1155/2021/4814250

**Published:** 2021-12-24

**Authors:** Ru Li, Xiao Liang, Fang Liu, Ziwei Zhou, Zhenzhen Zhang, Yongshen Lu, Peng Wang, Binrang Yang

**Affiliations:** ^1^Faculty of Physical Education, Shenzhen University, Shenzhen, China; ^2^Department of Sports Science and Physical Education, The Chinese University of Hong Kong, Hong Kong, China; ^3^Cardiac Rehabilitation Center, Fuwai Hospital, CAMS & PUMC, Beijing, China; ^4^Shenzhen Children's Hospital, Shenzhen, China

## Abstract

This study examined the mediating role of motor competence in the association between physical activity (PA) and quality of life (QoL) and the moderating role of age in the indirect relationship between PA and QoL in children with ADHD. Eighty-six children aged 6-12 years old (*M* age = 8.45, SD = 1.40, 17.4% girls) with the diagnosis of ADHD were recruited in this study. Participants wore a wGT3X-BT accelerometer on their wrist for seven consecutive days to measure PA. Motor competence was measured by the Test of Gross Motor Development-Third Edition (TGMD-3). Quality of life (QoL) was assessed by the parent-reported Chinese version of the Pediatric Quality of Life Inventory. MVPA was positively associated with object control skills but was not directly related to QoL. Using the bootstrapping method, the indirect effect of object control was found between MVPA and social functioning (0.10, 95%CI = [0.01, 0.21]), school functioning (0.09, 95%CI = [0.01, 0.18]), and overall QoL (0.07, 95%CI = [0.01, 0.16]), supporting the full mediation effect. Moderated mediation analysis further revealed that age strengthened the indirect effect from MVPA to social and school functioning via object control. Findings of this study indicated that MVPA is positively associated with object control skills, which in turn, is related to psychological aspects of QoL in children with ADHD. Age was found to moderate the indirect mediation paths. The findings may inform future expeditions on designing an effective intervention that helps to improve MC and QoL in children with ADHD.

## 1. Introduction

Attention deficit hyperactivity disorder (ADHD) is a prevalent neurodevelopmental disorder characterized by age-inappropriate inattention, impulsivity, and hyperactivity [1] that affects around 2.2%–7.2% of school age children worldwide [2]. Males are more commonly diagnosed with ADHD in children and adolescents, with the gender ratio ranging from 2 : 1 to 10 : 1 [3, 4]. Higher male-to-female ratios were observed in clinical versus population-based samples [5]. The Diagnostic and Statistical Manual of Mental Disorders (DSM–5) characterizes ADHD by three different subtypes, including predominantly hyperactive-impulsive subtype (ADHD-HI), predominantly inattentive subtype (ADHD-I), and a combined subtype (ADHD-C) [1]. Although ADHD is commonly diagnosed in childhood, the symptoms may maintain throughout adolescence and persist into adulthood in up to 70% of children with ADHD [6], which may produce negative consequences on their daily routine quality of life (QoL).

There is a growing realization that psychiatric comorbidity is the “rule rather than the exception” ([7], p. 1543) for children with ADHD. ADHD may experience significant difficulties that go beyond the core symptoms of ADHD in social incompetence, sleep problems, academic underachievement, poor motor proficiency, and executive dysfunction [8–12]. These negative outcomes may imply substantial deficits in various aspects of QoL, directly impairing physical and psychosocial well-being of children with ADHD. It is estimated that 50% of individuals with ADHD manifest developmental coordination disorder (DCD) in comparison with approximately 2-6% of the general population [13, 14]. A growing body of evidence supports the notion that children with DCD have lower overall health-related QoL compared to healthy counterparts [15–17]. According to the World Health Organization, QoL is defined as “an individual's perception of their position in life in the context of the culture and value systems in which they live and to their goals, expectations, standards and concerns” ([18], p. 1405). Specifically, QoL reflects one's subjective perception of the influence of life situation, including disease and treatment, on physical, psychological, and social functioning [19, 20]. QoL has become a major focus of outcome measures in child and adolescent health studies, particularly for those with developmental disorders who are vulnerable to physical and mental problems. There is emerging evidence showing that children with ADHD reported lower level of QoL compared with their typically developing (TD) peers [21]. Particularly, ADHD severely compromises children's physical functioning, psychosocial health, and general life quality, with increased symptom level resulting in worse QoL [20, 22]. There has been little discussion about the factors that are associated with QoL in children with ADHD [13]. Thus, it is imperative to understand the antecedents and potential mediators or moderators of various domains of QoL that may inform educators and healthcare to design effective interventions benefiting QoL.

Physical activity (PA) was observed to be positively related to a range of physical and mental health outcomes in youth [23]. Past reviews generally reported a significant positive association between PA and QoL in youth (Hedges′ *g* = 0.302) [24]. Specifically, moderate-to-vigorous PA (MVPA) is strongly correlated with QoL in children, implying that physically active children may experience improvement in QoL [25]. The latest WHO Guidelines on Physical Activity and Sedentary Behaviours recommends that children and adolescents living with disability should engage in at least 60 minutes of MVPA daily, including children and adolescents with ADHD [26]. Unfortunately, previous research measuring PA in children with ADHD have proven that they could not meet the minimum recommended criteria of 60 mins/day in MVPA [27, 28]. One recent study found that children with ADHD who engaged in increased amounts of PA reported higher QoL scores than those who were less physically active and sedentary [29]. It seems that increased time spent in MVPA may be a potential antecedent factor contributing to improved QoL in children with ADHD; however, the underlying mechanisms regarding possible mediators or moderators between PA and QoL in children with ADHD have not been established.

Motor competence (MC) refers to the degree of motor proficiency in performing fundamental movement skills (FMS) including both locomotor (e.g., jumping) and object control (e.g., catching) skills [30]. FMS competency is indispensable for children to master daily activities, which is commonly regarded as the essential building blocks for developing senior lifetime movement and context-specific motor skills from childhood to adulthood [31]. The fact is that motor impairments occur frequently in 30-40% children with neurodevelopmental disorders [10]. Specific fine and gross motor difficulties were observed in children with ADHD [32]. The relationship between MC and PA participation has gained strong empirical support, and several studies have proposed their bidirectional associations in TD children. For example, greater motor proficiency in youth may predict MVPA participation and health throughout lifespan [33]; reversely, increased PA may enhance the level of mastery of motor skills [34]. Nevertheless, there is a paucity of evidence supporting the direct link between PA and MC in children with ADHD. Intervention studies indicated that physical activity intervention had positive effect on motor proficiency in children with ADHD [35, 36].

Additionally, poor MC was found to be connected with impaired psychosocial health in children [37]. A recent study showed that children with higher gross motor competence scores perceived higher health-related QoL [38]. Based on the conceptual model proposed by Blair [39], health-related behaviors (e.g., PA, diet, and alcohol consumption) influence various components of fitness (e.g., muscular, cardiorespiratory, motor, and metabolic components), which in turn, affect health-related outcomes. Theoretically, it is reasonable to assume the indirect association between PA and health-related QoL through the mediation of motor components of fitness. Therefore, it could be speculated that higher levels of MVPA were related to greater performance on MC, which, in turn, was associated with better QoL in children. Causalities among these variables were not established, leaving unclear whether MC could be considered as outcome of MVPA and antecedent factor predicting QoL. Stodden et al. [40] presented a theoretical framework where PA and MC exhibited varied bidirectional associations from early to middle childhood. In alignment with this theoretical framework, Lima et al. [41] found reciprocal longitudinal relationship between PA and MC and further implied that the strength of their changes across childhood and adolescence. Furthermore, age was assumed to be a potential moderator in the link between PA and MC as another longitudinal study found that children at young age (2-6 years old) demonstrated diverse levels of PA and MC that were unrelated and suggested the relationship between PA and MC evolves as children age [42]. One previous review found that age was negatively associated with QoL in children and adolescents with ADHD [20]. To date, no previous studies examined whether age as a moderator in the indirect relation between MVPA and QoL via MC. In this context, exploring the potential antecedents of, and pathways for, improved QoL in children with ADHD is of paramount importance.

**Figure 1:**
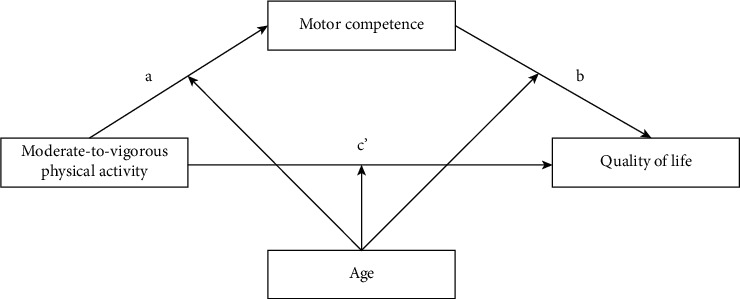
The proposed moderated mediation model.

The purpose of this study was twofold: (a) to investigate whether MC would mediate the association between MVPA and different domains of QoL in children with ADHD and (b) to test whether the direct and indirect relations between MVPA and QoL via MC were moderated by age. The proposed model is illustrated in Figure 1.

## 2. Materials and Methods

### 2.1. Participants

Children aged 6-12 years old were recruited from the outpatient clinics of Children' Healthcare & Mental Health Center at Shenzhen Children's Hospital. Inclusion criteria include (1) ADHD diagnoses based on the criteria of DSM-5 [1], (2) having a full IQ of more than 70 measured by the Chinese Wechsler Intelligence Scale for Children, fourth edition [43], (3) having no comorbid axis-I psychiatric disorders or a history of major neurological diseases, and (4) having no other medical conditions that limited their physical activity capacities (e.g., asthma and cardiac disease). A total of 86 medication-naïve patients with ADHD were recruited, and their diagnoses were confirmed by two experienced psychiatrists who utilized the standard structured interview according to the Schedule for Affective Disorders and Schizophrenia for School-Age Children-Present and lifetime Version [44, 45]. All participants were full-time students enrolled in regular schools.  Table 1 describes the characteristics of the participants.

### 2.2. Measures

#### 2.2.1. Physical Activity

Physical activity data used for analysis were time spent in MVPA, which was objectively measured by asking the participants to wear the Actigraph wGT3X-BT accelerometer device. Children were required to wear the devices on the nondominant wrist for seven consecutive days and were instructed to only take it off when they were taking baths or swimming. Sampling rate was set to 60 Hz. Data in raw format as GT3X file was converted to raw csv file format for signal processing in *R* using GGIR package version 2.4-0 [46]. Signal processing in GGIR involves the detection of nonwear time and the calculation of the average magnitude of dynamic acceleration corrected for gravity (ENMO). A valid day was defined as accelerometer wearing for 100% during the daytime. Participants were excluded if they were of less than four valid days of wear. The MVPA cut point was classified as 100 mg (MVPA threshold parameter = *c* (100)), which had been validated for wrist wearing in children [47]. MVPA values were computed by averaging MVPA metrics of all valid days for each participant.

#### 2.2.2. Motor Competence

The motor competence was assessed based on the standardized protocols described in the Test of Gross Motor Development-Third Edition [48]. The TGMD-3 has been validated in China with satisfied reliability, which provides an objective instrument evaluating basic motor skills for Chinese children [49]. It assesses the performance of six locomotor and seven object control skills with a total raw score of 100. In accordance with previous study [50], three locomotion tasks (i.e., running, skipping, and jumping) and three ball skill tasks (i.e., catching, overhand throw, and underhand throw) were selected to represent locomotion and object control capacities, respectively. Before the assessment of each skill, a trained researcher first demonstrated the skill, and the participants were allowed one practice trial. Then, the participants performed two formal trials that were observed and coded by an experienced rater. Six fundamental movement skills of all participants were individually videotaped using an iPad for later analysis by another rater. The two independent raters were trained, and they had to exceed an interrater agreement (IOA) score of 85% before conducting formal assessments. Each skill was assessed based on 3-5 performance criteria that are scored 1 point (present) or 0 point (absent) using process-oriented checklists. The scores rated from two attempts of each skill were summed to produce a total score of locomotion and object control. The two independent raters evaluated all trials of each skill. The interrater agreement was strong (ICC = 0.88, 95%CI = [0.68, 0.95]). The disagreement between the two raters was discussed and reassessed to decide the final score.

#### 2.2.3. Quality of Life

The 23-item Pediatric Quality of Life Inventory (PedsQLTM Generic Core Scales) [51] was used to assess health-related QoL in children and adolescents aged 2-18 years old. The Standard Versions of Parent Proxy-report for Young Child (5-7 years of age) and Child (8-12 years of age) were used considering different age groups of the participants. The PedsQL includes four subscales (physical, emotional, social, and school), of which physical health summary score was computed by averaging 8 items of physical functioning (same as the physical functioning scale), and the psychological health summary score was computed by averaging 15 items of emotional, social, and school functioning subscales. Parent proxy-reported scores were collected in this study. Scores range from 0 to 100, with higher scores suggesting better QoL. This study used the Chinese version of the PedsQLTM Core (Standard), which has been psychometrically evaluated and well validated [52]. The internal consistency of each subscale was reported to be acceptable in this study (physical : *α* = 0.82; emotional : *α* = 0.77; social : *α* = 0.86; school: *α* =0.73).

#### 2.2.4. Confounders

For testing that meditation effect of MC between MVPA and QoL, confounders included children's age, gender, and BMI; ADHD subtype (ADHD-I, ADHD-C, and ADHD-HI); and ADHD symptom severity assessed by Chinese version of the Swanson, Nolan, and Pelham Version IV Scale-parent form (SNAP-IV [53]).

### 2.3. Procedure

This study was approved by the research ethics committees of Shenzhen Children's Hospital, Shenzhen, China (Approval ID: 202010302). Informed consent form was obtained from parents in accordance with the Declaration of Helsinki. Participants visited the lab twice individually with their parents. For the first time, their weight and height were measured, and body mass index (BMI) was calculated by dividing body mass (kg) by stature squared (m^2^). Subsequently, they finished the TGMD-3 before they were provided the accelerometer. Their parents completed the questionnaire of PedsQL and SNAP-IV. The participants visited the lab again to return the accelerometer after wearing it for seven days.

### 2.4. Data Analysis

Descriptive statistics were calculated for MVPA, motor competence score, and QoL. Bivariate correlations among the variables were calculated. The mediating and moderating effects were analyzed using bootstrapping regression models with PROCESS function of bruce*R* package 0.7.0 in *R* [54]. PROCESS models developed by Hayes [55] were applied to test the mediating effect of motor competence as well as the moderating effect of age on the direct and indirect links between MVPA and QoL after controlling for potential confounders (i.e., gender, BMI, ADHD subtype, and ADHD symptom severity). All continuous variables were mean centered, and bootstrapping confidence intervals (CIs) utilizing 5000 resamples were used for model estimations.

## 3. Results

### 3.1. Preliminary Analyses

Missing data was absent for each variable. All participants had valid accelerometery data of at least four days. Descriptive statistics, skewness, kurtosis, and correlations among the study variables are demonstrated in Table 2. The data was examined for normality of distribution by skewness (∣skewness | <3) and kurtosis (∣kurtosis | <10) [56]. MVPA was significantly associated with object control skills, but was not directly associated with all dimensions of QoL. Object control was positively associated with social functioning, school functioning, and total score of QoL.

### 3.2. Testing for Mediation Effect

PROCESS model 4 developed by Hayes [55] was used to test the mediation effect of motor skills (locomotion, object control) between MVPA and QoL. The structure and the results of the model applied are reported below. MVPA was the focal predictor (*F*), MC (object control, locomotion) was the proposed mediator (*M*), and age, gender, BMI, ADHD subtype, and ADHD severity were the covariates (*C*). *F*, *M*, and *C* were included as predictors in a bootstrapping regression analysis predicting the QoL. The following model was tested for each dimension of QoL:

(1) $$\begin{array}{c} \text{QoL}=a+b1\text{ age}+b2\text{ gender}+b3\text{ BMI}+b4\text{ ADHD subtype}+b5\text{ ADHD severity}+b6\text{ MVPA}+b7\text{ MC} \end{array}$$

The regression results showed that only object control mediates the relation between MVPA and quality of life. Specifically, MVPA was positively related to object control, which was, in turn, positively associated with social functioning, school functioning, and total score of QoL, respectively. The mediation effects of locomotion between MVPA and various domains of QoL were not found.  Table 3 displays the path coefficients of the mediation effects. These results support that object control fully mediated the association between MVPA and social functioning, school functioning, and overall QoL, and the mediation effect accounted for 15%-22% of the total effect of MVPA on these outcomes. The regression results for significant mediation effects were reported in Table 4.

### 3.3. Moderated Mediation Effect

PROCESS model 59 developed by Hayes [55] was used to further examine the direct and indirect relationships between MVPA and QoL via object control moderated by age. The analysis equation changed as follows:

(2) $$\begin{array}{c} \text{QoL}=a+b1\text{ gender}+b2\text{ BMI}+b3\;A\text{DHD subtype}+b4\text{ ADHD severity}+b5\text{ age}+b6\text{ MVPA}+b7\left[\text{MVPA}\times \text{age}\right]+b8\text{ MC}+b9\left[\text{MC}\times \text{age}\right] \end{array}$$

**Figure 2:**
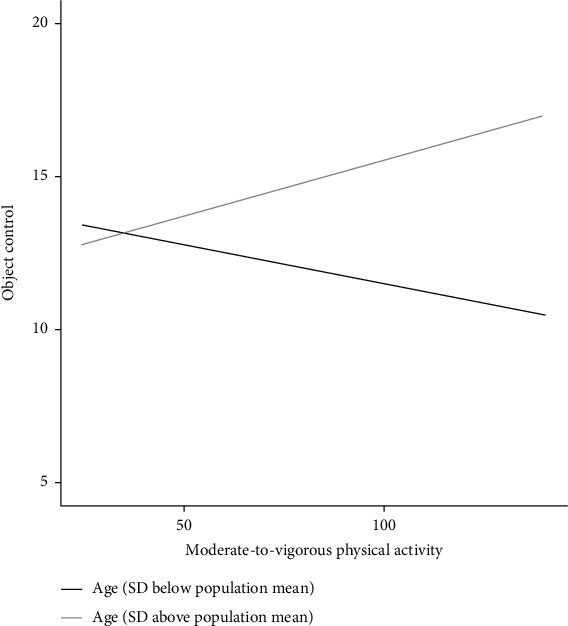
Predicted object control against MVPA via the moderation of age.

With age as a moderator in the regression model, the indirect effect on overall QoL turns to be nonsignificant. The results of parameters for social and school functioning are shown in Table 5. Model 1 of Table 5 shows that the interaction effects (product) of MVPA and age had a significant positive association with object control (*F* = 7.91, *p* = .006). Simple slope test demonstrated that MVPA was positively associated with object control for older children with ADHD (*b*_simple_ = 0.06, *p* < .001), while MVPA yielded a nonsignificant association with object control for younger children with ADHD (*b*_simple_ = −0.01, *p* = .86). Simple slope tests demonstrated that age moderated the relation between MVPA and object control; with the older age of the children, the stronger association between MVPA and object control was reported.  Figure 2 shows a clear demonstration of the moderating role of age, separately for different age groups (one SD below and one SD above the mean, respectively). It means that stronger association between MVPA and object control was reported in children aged one SD above the mean (9.85 years) compared to those aged one SD below the mean (7.05 years).

Additionally, model 2 of Table 5 illustrated there was a significant main effect of object control on social and school functioning, but these effects were not moderated by age (all *p* > .05). Similarly, age did not significantly moderate the association between MVPA and social and school functioning) (all *p* > .05).

The bias-corrected percentile bootstrap analysis further indicated that the indirect effect of MVPA on social and school functioning via object control was moderated by age. Specifically, the indirect effects of MVPA on social and school functioning were significant for older children with ADHD (social functioning: *b* = 0.05, SE = 0.03, 95%CI_boot_ = [0.01, 0.11]; school functioning: *b* = 0.03, SE = 0.02, 95%CI_boot_ = [0.01, 0.07]). However, the indirect effect was not significant for younger children with ADHD (social functioning: *b* = −0.01, SE = 0.02, 95%CI_boot_ = [−0.05, 0.04]; school functioning: *b* = −0.01, SE = 0.02, 95%CI_boot_ = [−0.06, 0.03]). Results showed that object control mediated the effect of MVPA on QoL, but a younger age weakened the mediating effect of object control.

## 4. Discussion

This study is aimed at investigating whether MC mediated the relationship between MVPA and QoL in children with ADHD, and whether age played a moderating role in the direct and indirect relations between MVPA and QoL. The findings partially support the indirect effects of object control skills on the relationship between MVPA and psychological health and overall QoL. Furthermore, age moderated the indirect effects of MVPA on psychological health through object control skills.

Despite that the significant mediating effect of MC was found, the direct association between MVPA and QoL was not found in our study. This was inconsistent with one recent study, demonstrating that a greater frequency of PA contributes to a higher level of QoL in children with ADHD [29]. The possible explanation of this finding may be related to the dose of daily MVPA in children with ADHD. One previous review concluded that increased PA would result in better QoL in children and adolescents [24], but this association was only proved in the TD group. More importantly, they proposed a dose-response relationship between PA and QoL manifesting that an increased PA level by over 2 hours/day could achieve a clinically meaningful change in QoL [24]. In our study, the participants in our study attended averagely 85 minutes of MVPA daily. Although they met the minimum recommended PA guidelines of 60 mins/day, it is still perhaps lacking PA volume that failed to initiate improvements on QoL, both physically and psychologically. Additionally, although habitual PA per se may be important for various domains of QoL, it may not promote QoL directly. Specific types or quality of activity experiences, rather than overall quantity of PA, may be more related to QoL. Our findings that PA was indirectly associated with QoL via MC may further confirm this speculation. Furthermore, Gallego-Mendez and colleagues' study [20] used child-and-parent reported questionnaire in assessing their PA behaviors, which probably caused measurement bias that could explain the differences from our objective assessments. More studies exploring the relationships between PA and QoL in a larger sample of children with ADHD are needed.

MVPA was found to be positively related to MC, which is consistent with our hypothesis and is supported by previous studies. More physically active children were more likely to master high levels of MC [57]. Unfortunately, children with ADHD are likely to be restricted in participation in various daily activities at school [58], at home [59], and at other community settings [60], which may lead to reduced opportunities for them to acquire motor skills. Although children with ADHD were observed to be clumsy when performing motor skills [10], previous intervention studies indicated that increased levels of PA intervention showed beneficial effects on the development of MC and alleviation of ADHD symptoms in children with ADHD [61]. Our cross-sectional evidence confirmed that enhanced motor skills could be associated with elevated MVPA. Additionally, simply object control skills, rather than locomotion skills, were positively associated with social and school functioning and general level of QoL. This is aligned with previous findings in children with DCD that ball skills (aiming and catching) were significant related to school aspects of QoL [62]. Object control capacities, which were evaluated through catching and throwing tasks, are more likely to be involved in some specified types of team sports or goal-directed games/activities. In the setting of team sports/games, object control capacity of an individual could be observed from his/her performance in specific motor skills, such as passing, receiving, and shooting in ball games [63]. The proficiency in these motor skills may provide the foundation for peer relationships, as well as physical and psychosocial growth, which may account for the salient role of object control versus locomotion in relation to QoL.

Object control skills significantly mediate the associations between MVPA and psychological functioning (i.e., social and school) and general level of QoL. Previous studies found that exercise interventions with high levels of PA might improve both motor skills and physical health of QoL in children with ADHD [64]. However, the mechanisms underlying the relationships among PA, MC, and QoL remain unclear. Our findings provide preliminary evidence regarding the mechanisms, suggesting that ADHD children who are more physically active may affect their health-related QoL through improved motor proficiency, which interacts to result in positive downstream effects on physical and psychological functioning. Since the cerebellum is responsible for motor control, the abnormalities in the cerebellum identified in individuals with ADHD may cause delays in motor development [10]. Higher levels of PA boost the activation of the cerebellum, which may explain the exercise-induced MC improvements in children with ADHD. Furthermore, motor performance is linked to the mental representation of activity and other variables such as cognitive functions, social relationships, and physical and psychological health [10]. It is not necessary that children with ADHD could benefit directly from increased MVPA level, unless their MC was adequately improved through activity participation. Kaminsky et al. [65] proposed that health-related physical fitness including MC seems to be more important than PA in relation to health outcomes. Practically, this study informs future challenges to target on MC-oriented PA interventions with the efforts of promoting and maintaining adequate motor skills, instead of simply focusing on PA intensities and frequencies.

Lastly, our findings indicate that age could moderate the relation between MVPA and object control skills as well as indirect path between MVPA and QoL. The relationship between MVPA and MC turns to be stronger in older children in comparison to younger children with ADHD. Plausible explanation could be that as ADHD children getting older, physical activity participation is more facilitative for shaping motor skills. These findings, to some extent, confirm the previous speculations that the relationship between PA and MC evolves as children age [42]. Additionally, the moderating role of age in the indirect path from MVPA to social and school functioning implies that older children tend to benefit from MVPA on their psychological functioning through gaining proficiency in object control capacities. We encourage educators to consider age-appropriate strategies with the incorporation of more ball skills training in children's daily activities as they grow up. In other words, it is never too late for children to become physically active as it benefits them with different mechanisms.

The current study had several strengths (e.g., objectively measuring PA by accelerometer and utilizing TGMD-3 to evaluate MC). However, several limitations should be addressed. First, although it was recommended that the appropriate sample size to test a medicating effect using the bootstrapping method should be at least ranging from 50 to 100 participants [66], the relatively small sample size in our study may result in low statistical power to detect existing associations. Second, the perception of QoL was simply parent-reported. As the previous review found substantial variations in the parent-child agreement in their perception of QoL [20], and children and adolescents with ADHD tend to report their QoL less negatively than their parents [22], this may be a source of bias affecting the results. Future studies should consider exploring both child- and parent-rated QoL. Third, TGMD-3 is appropriate for evaluating children's basic motor skills aged 3-10 years old. This study included a small percentage (5.8%) of participants older than 10 years old, whose performance might reach the ceiling effect of the TGMD-3 scale. Their true level of motor skill might not have been measured accurately, which probably causes bias to the moderating effect of age. Lastly, due to the great unbalance of gender distribution of ADHD itself, the moderating effect of gender was not explored.

## 5. Conclusion

Although further replication and extensions are necessary, the present study is an important step in unpacking how MVPA relates to various aspects of QoL of children with ADHD. Since MC served as one potential mechanism by which MVPA were correlated to QoL, it remains important to address motor impairment directly or intervene in a manner that promote the impact of MVPA on QoL. Our results may inform future expeditions of how nonpharmacological intervention (e.g., PA intervention) may help to improve MC, and therefore, QoL in children with ADHD. Particularly, age-appropriate strategies with increased MC-oriented intervention could help children with ADHD maximize benefits on health-related outcomes.

## Figures and Tables

**Table 1:** Participants' characteristics.

Characteristics	*N* = 86 mean (SD)
Age	8.45 (1.40)
Gender (boys/girls)	71/15
Height (cm)	131.46 (9.40)
Weight (kg)	29.56 (8.98)
BMI	16.77 (3.00)
ADHD subtype	
Inattention (%)	51 (59.3%)
Hyperactivity (%)	5 (5.8%)
Combined (%)	30 (34.9%)
Comorbidities	
Oppositional defiant disorder (%)	17 (19.8%)
Obsessive compulsive disorder (%)	2 (2.3%)
Tourette syndrome (%)	5 (5.8%)
Enuresis (%)	3 (3.5%)
Encopresis (%)	1 (1.2%)

**Table 2:** Descriptive statistics and bivariate correlations.

Variable	M	SD	Skewness	Kurtosis	1	2	3	4	5	6	7	8	9	10
1. MVPA	85.17	30.52	0.29	-0.19	—									
2. Locomotion	14.21	3.33	-0.53	-0.42	0.16	—								
3. Object control	13.08	3.09	-0.22	0.16	0.25^∗^	0.43^∗∗^	—							
4. TGMD total	27.29	5.35	-0.20	-0.57	0.29^∗^	0.85^∗∗^	0.84^∗^	—						
5. pH	70.28	17.16	-0.48	0.18	-0.10	0.10	0.16	0.10	—					
6. EF	65.64	17.27	0.10	-0.87	-0.06	-0.07	-0.04	-0.06	0.39^∗∗^	—				
7. SF	67.91	18.32	-0.00	-0.79	-0.08	0.03	0.28^∗^	0.18	0.53^∗∗^	0.39^∗∗^	—			
8. SCF	50.14	13.74	0.47	0.58	-0.06	0.01	0.26^∗^	0.19	0.55^∗∗^	0.41^∗∗^	0.56^∗∗^	—		
9. PSY	61.23	13.27	0.22	0.07	-0.05	0.03	0.20	0.12	0.61^∗∗^	0.77^∗∗^	0.83^∗∗^	0.79^∗∗^	—	
10. QoL	63.60	12.92	-0.17	-0.46	-0.06	0.01	0.22^∗^	0.12	0.86^∗∗^	0.68^∗∗^	0.78^∗∗^	0.76^∗∗^	0.93^∗∗^	—

Note. ^∗^*p* < 0.05; ^∗∗^*p* < 0.01. MVPA: moderate-to-vigorous physical activity; TGMD: test of gross motor development; pH: physical health summary; EF: emotion functioning; SF: social functioning; SCF: school functioning; PSY: psychological health summary; QoL: quality of life.

**Table 3:** Bootstrap analysis summary showing the indirect effects of MVPA on QoL via object control and locomotion.

Path	*a* path coefficient	*b* path coefficient	*c*′ path coefficient (direct effect)	Indirect effect (*a* × *b*)			
				Point estimate	SE	*Z*	Boot 95% CI
MVPA-OC-PH	0.31^∗∗^	0.20	-0.07	0.06	0.04	1.69	[-0.01, 0.14]
MVPA-OC-EF	0.31^∗∗^	-0.05	0.04	-0.01	0.04	-0.36	[-0.10, 0.06]
MVPA-OC-SF	0.31^∗∗^	0.31^∗∗^	-0.05	0.10	0.05	1.88	[0.01, 0.21]^∗^
MVPA-OC-SCF	0.31^∗∗^	0.28^∗^	-0.05	0.09	0.04	1.97	[0.01, 0.18]^∗^
MVPA-OC-PSY	0.31^∗∗^	0.22	-0.03	0.07	0.04	1.57	[-0.01, 0.16]
MVPA-OC-QOL	0.31^∗∗^	0.24^∗^	-0.05	0.07	0.04	1.78	[0.01, 0.16]∗
MVPA-LM-PH	0.27^∗^	0.01	-0.01	0.01	0.04	0.01	[-0.08, 0.08]
MVPA-LM-EF	0.27^∗^	-0.06	0.04	-0.02	0.04	-0.45	[-0.11, 0.04]
MVPA-LM-SF	0.27^∗^	-0.02	0.05	-0.01	0.03	-0.13	[-0.08, 0.06]
MVPA-LM-SCF	0.27^∗^	0.02	0.03	0.01	0.04	0.14	[-0.09, 0.07]
MVPA-LM-PSY	0.27^∗^	-0.03	0.05	-0.01	0.04	-0.28	[-0.10, 0.05]
MVPA-LM-QOL	0.27^∗^	-0.02	0.03	-0.01	0.04	-0.13	[-0.09, 0.06]

Note. ^∗^*p* < .05; ^∗∗^*p* < .01. Standardized path coefficients are presented. MVPA: moderate-to-vigorous physical activity; OC: object control; LM: locomotion; PH: physical health summary; EF: emotional functioning; SF: social functioning; SCF: school functioning; PSY: psychological health summary; QOL: quality of life.

**(a):** 

Predictors	Model 1 (SF)			Model 2 (object control)			Model 3 (SF)		
	Standardized coefficient	Unstandardized coefficient	SE	Standardized coefficient	Unstandardized coefficient	SE	Standardized coefficient	Unstandardized coefficient	SE
Age	0.07	0.86	1.50	0.37^∗∗^	0.87^∗∗^	0.26	-0.05	-0.62	1.54
Gender	-0.14	-6.70	5.36	-0.20	-1.72	0.90	-0.08	-3.86	5.30
BMI	-0.14	-0.82	0.68	0.11	0.12	0.12	-0.17	-1.01	0.66
ADHD subtype	0.09	1.80	2.18	0.06	0.21	0.37	0.08	1.44	2.10
ADHD severity	-0.40^∗∗^	-0.86^∗∗^	0.26	-0.14	-0.06	0.04	-0.36	-0.77^∗∗^	0.25
MVPA	0.04	0.02	0.07	0.31^∗∗^	0.03^∗∗^	0.01	-0.05	-0.03	0.07
Object control							0.31^∗∗^	1.70^∗^	0.63
*R* ^2^	0.15			0.24			0.22		

**(b):** 

Predictors	Model 1 (SCF)			Model 2 (object control)			Model 3 (SCF)		
	Standardized coefficient	Unstandardized coefficient	SE	Standardized coefficient	Unstandardized coefficient	SE	Standardized coefficient	Unstandardized coefficient	SE
Age	0.08	0.77	1.11	0.37^∗∗^	0.87^∗∗^	0.26	-0.02	-0.20	1.15
Gender	0.06	1.91	3.98	-0.20	-1.72	0.90	0.12	4.04	3.93
BMI	-0.07	-0.29	0.51	0.11	0.12	0.12	-0.10	-0.42	0.50
ADHD subtype	0.06	0.82	1.62	0.06	0.21	0.37	0.04	0.58	1.58
ADHD severity	-0.31^∗^	-0.48^∗^	0.19	-0.14	-0.06	0.04	-0.27^∗^	-0.42^∗^	0.19
MVPA	0.04	0.02	0.05	0.31^∗∗^	0.03^∗∗^	0.01	-0.05	-0.02	0.05
Object control							0.28∗	1.12∗	0.47
*R* ^2^	0.09			0.24			0.15		

**(c):** 

Predictors	Model 1 (QOL)			Model 2 (object control)			Model 3 (QOL)		
	Standardized coefficient	Unstandardized coefficient	SE	Standardized coefficient	Unstandardized coefficient	SE	Standardized coefficient	Unstandardized coefficient	SE
Age	-0.05	-0.45	1.08	0.37^∗∗^	0.87^∗∗^	0.26	-0.14	-1.25	1.13
Gender	-0.16	-5.32	3.82	-0.20	-1.72	0.90	-0.12	-3.86	3.85
BMI	0.00	0.01	0.49	0.11	0.12	0.12	-0.02	-0.10	0.48
ADHD subtype	0.15	2.05	1.57	0.06	0.21	0.37	0.14	1.85	1.54
ADHD severity	-0.42^∗∗∗^	-0.63^∗∗∗^	0.18	-0.14	-0.06	0.04	-0.38^∗∗^	-0.58^∗∗^	0.18
MVPA	0.03	0.01	0.05	0.31^∗∗^	0.03^∗∗^	0.01	-0.05	-0.02	0.05
Object control							0.24^∗^	0.93^∗^	0.46
*R* ^2^	0.14			0.24			0.18		

Note. ^∗^*p* < .05; ^∗∗^*p* < .01, ^∗∗∗^*p* < .001. Both standardized and unstandardized regression coefficients are presented. MVPA: moderate-to-vigorous physical activity; SF: social functioning; SCF: school functioning; QOL: quality of life.

**(a):** 

Predictors	Model 1 (object control)		Model 2 (SF)	
	Unstandardized coefficient	SE	Unstandardized coefficient	SE
Gender	-1.42	0.88	-3.86	5.33
BMI	0.22	0.12	-0.60	0.70
ADHD subtype	0.32	0.36	2.06	2.10
ADHD severity	-0.07	0.04	-0.84^∗∗^	0.25
MVPA	0.03^∗^	0.01	-0.06	0.07
Age	0.77^∗∗^	0.25	-1.07	1.60
MVPA×age	0.02^∗∗^	0.01	0.08	0.05
Object control			1.40^∗^	0.66
Age×object control			0.34	0.44
*R* ^2^	0.31		0.26	

**(b):** 

Predictors	Model 1 (object control)		Model 2 (SCF)	
	Unstandardized coefficient	SE	Unstandardized coefficient	SE
Gender	-1.42	0.88	4.56	4.04
BMI	0.22	0.12	-0.39	0.54
ADHD subtype	0.32	0.36	0.53	1.61
ADHD severity	-0.07	0.04	-0.41^∗^	0.19
MVPA	0.03^∗^	0.01	-0.01	0.06
Age	0.77^∗∗^	0.25	-0.02	1.23
MVPA×age	0.02^∗∗^	0.01	0.01	0.04
Object control			1.08∗	0.51
Age×object control			-0.16	0.34
*R* ^2^	0.31		0.15	

Note. ^∗^*p* < .05; ^∗∗^*p* < .01. Unstandardized regression coefficients are presented. MVPA: moderate-to-vigorous physical activity; SF: social functioning; SCF: school functioning.

## Data Availability

All data generated or analyzed during this study were included in the article.
